# Interleukin-1 Beta (IL1B) and Nerve Growth Factor (NGF): Key Players in Rabbit Reproductive Regulation

**DOI:** 10.3390/ijms252010986

**Published:** 2024-10-12

**Authors:** Gabriella Guelfi, Cecilia Dall’Aglio, Antonello Bufalari, Francesca Mercati, Polina Anipchenko, Camilla Capaccia, Paolo Cocci, Francesco Alessandro Palermo, Gabriele Acuti, Alessandro Troisi, Daniele Tomassoni, Cristiano Boiti, Massimo Zerani, Margherita Maranesi

**Affiliations:** 1 Department of Veterinary Medicine, University of Perugia, Via San Costanzo 4, 06126 Perugia, Italy; gabriella.guelfi@unipg.it (G.G.); cecilia.dallaglio@unipg.it (C.D.); apsvet93@gmail.com (P.A.); camilla.capaccia@dottorandi.unipg.it (C.C.); gabriele.acuti@unipg.it (G.A.); boiti.cristiano@gmail.com (C.B.); massimo.zerani@unipg.it (M.Z.); margherita.maranesi@unipg.it (M.M.); 2 School of Bioscience and Veterinary Medicine, University of Camerino, Via Gentile III da Varano, 62032 Macerata, Italy; paolo.cocci@unicam.it (P.C.); francesco.palermo@unicam.it (F.A.P.); alessandro.troisi@unicam.it (A.T.); daniele.tomassoni@unicam.it (D.T.)

**Keywords:** IL1B, IL1R1, PGE2, PGF2α, uterus, testis, prostate, seminal vesicles, seminal plasma, NGF

## Abstract

Several seminal plasma components, besides NGF, are implicated as ovulation-inducing factors in mammals. This study investigated the IL1B and its receptor IL1R1 in the testis (T), male accessory glands, prostate (P) and seminal vesicles (SV), and uterus (U) of adult rabbits using immunohistochemistry (IHC) and quantitative reverse transcription PCR (RT-qPCR). We also assessed the presence of IL1B in seminal plasma through Western blotting (WB) and examined the interaction between IL1B and NGF in vitro by measuring their production with enzyme-linked immunosorbent assay (ELISA) in the presence of NGF and IL1B alone or with their respective receptor antagonists. IHC revealed IL1B system expression in all reproductive organs studied, with IL1B and IL1R1 localized to the germinative epithelium of the T and the epithelial cells of the accessory glands and U. IL1B gene transcript levels were significantly higher (*p* < 0.01) in the P and SV compared to the T, while IL1R1 levels were significantly higher (*p* < 0.001) in the P compared to the other tissues, while IL1R1 levels were three times higher (*p* < 0.001) in the P. WB confirmed the presence of IL1B in seminal plasma with a 30–35 kDa band. The in vitro study demonstrated that IL1B increased (*p* < 0.05) basal NGF production in the U, whereas NGF had no effect on IL1B production. These findings provide evidence of the expression of the IL1B/IL1R1 system in both male and female rabbit reproductive tracts and suggest that IL1B in seminal plasma may influence uterine endocrine activity. The results propose a potential role for IL1B in ovulation, in conjunction with NGF, supporting that ovulation may involve inflammatory-like processes.

## 1. Introduction

There is growing evidence that seminal plasma components are involved in both male and female reproductive processes. In addition to NGF, a well-known ovulation-inducing factor, other cytokines in seminal plasma can stimulate the female reproductive tract and lead to ovulation, thereby improving reproductive success [1].

IL1, the first cytokine to be discovered, plays a crucial role in immune and inflammatory processes and may have key physiological importance in the female and male reproductive systems [2,3]. Nevertheless, cytokines can become extremely detrimental to gonadal function when their levels are higher than normal, as in inflammatory situations [4].

There are two main IL1s, IL1A and IL1B. When secreted, IL1B undergoes proteolytic processing from an inactive 35 kDa precursor to an active 17 kDa molecule. It is generally accepted that IL1A, the other isoform, acts as an autocrine growth factor or as a mediator of direct cell-to-cell communication. Although IL1A can be released, IL1A prefers to remain cell-associated [5].

IL1B signaling occurs through the interleukin-1 receptor type 1 (IL1R1)/interleukin 1 receptor accessory protein (IL1R3) heterodimer, triggering pathways like mitogen-activated protein kinase (MAPK) pathway or the nuclear factor-kappa B (NF-kB), which activate transcription of numerous genes, including IL1B, too [6,7]. IL1B is synthesized in the pregnant rabbit cervix, largely by leukocytes [8] and it is a potent regulator of prostaglandin (PG) synthesis [6]. It also stimulates the production of cytokines like Interleukin-6 (IL6) and tumor necrosis factor (TNF), which further enhance PG synthesis [9]. IL1B’s role in PG production and ovulation is well-established, promoting Cyclooxygenase-2 (COX2) synthesis and inducing PGs in granulosa cells across various species [10]. It also enhances phospholipase A2 activity in the ovary, boosting PG production and stabilizing its mRNA [11].

Research on rabbits first described inflammatory responses in females post-mating, revealing a leukocytic influx in the U triggered by seminal plasma but not by sterile sperm [12]. Although coitus-induced ovulation is mediated by neuroendocrine mechanisms in rabbits, the role of seminal plasma as an ovulation-inducing factor is still debated question [13,14].

This research studied how IL1B was involved in male–female rabbit reproductive crosstalk. The investigational approach included two steps. The first phase (in males) aimed to investigate using RT-qPCR the expression levels of IL1B and its related receptor IL1R1 in rabbit male reproductive tissues, T, P, and SV, and to detect, using IHC, IL1B and IL1R1 protein location in male reproductive tissues. At this stage, circulating IL1B protein was assessed in seminal plasma using WB to investigate whether seminal plasma transported IL1B protein. In the second step (in females), based on a hypothetical functional role of IL1B in the rabbit U, the expression of IL1R1 and the co-receptor IL1R3 was investigated using RT-qPCR. IHC evaluated the IL1R1 uterine tissue location. Finally, an ex vivo rabbit uterine model was used to investigate the impact of molecules such as IL1B, IL1R1 antagonist, Tropomyosin receptor kinase A (TRKA) Inhibitor, p75 neurotrophin receptor (p75NTR) Inhibitor, and COX Inhibitor on the production of NGF, PGF2α, and PGE2. The secretion of NGF, PGF2α, and PGE2 in the tissue culture medium was analyzed through ELISA.

## 2. Results

### 2.1. IL1B and IL1R1 Male Tissue Gene Expression

The 260/280 RNA ratio was 1.95, while the 260/230 ratio was 2.1, indicating the high purity of RNA preparation in each sample. The yield of total RNA was not significantly different in the samples. Minimal variations in total RNA amount were adjusted in reverse transcription (RT) using a fixed RNA input. QPCR data of target genes of different tissues were examined using the normalized value 2^ −(Cq target gene − Cq reference gene).

**Figure 1:**
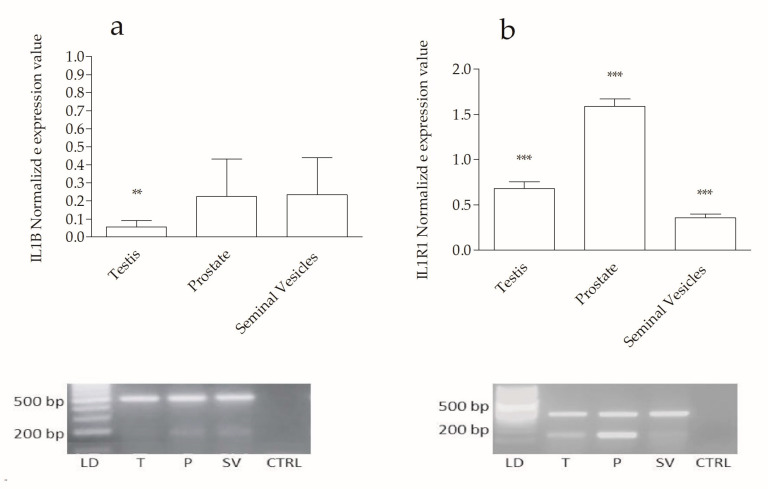
QPCR normalized expression values. The normalized gene expression values for IL1B (**a**) show a statistically significant upregulation in testis (T) (**, *p* < 0.01) compared to prostate (P) and seminal vesicles (SV). No significant differences in IL1B levels were observed between P and SV (*p* > 0.05). Regarding IL1R1 (**b**), normalized mRNA expression levels reveal significant differences (***, *p* < 0.001) between T, P, and SV. The bars above the histograms represent the standard error. Figure 1 below the graphs shows the results from the 2% agarose gel electrophoresis. In the gel images, the first lane (left) contains a 50 bp DNA ladder (LD), followed by lanes with the qPCR amplicons for P, T, and SV. The last lane (right) shows the qPCR negative control (CTRL). (**a**) shows the gel of IL1B (183 bp) and 18S (489 bp) qPCR amplicons from P, T, and SV, while (**b**) shows the gel for IL1R1 (137 bp) and 18S (489 bp) amplicons from the same samples.

The level of the IL1B gene in rabbit P and SV tissues was statistically significantly higher when compared with the T (*p* ˂ 0.01). T exhibited a lower IL1B transcript level in comparison with the accessory genital glands (Figure 1a). The gene expression levels of IL1R1 in T, P, and SV tissues, compared with each other, showed highly significant differences (*p* ˂ 0.001). IL1R1 in P appeared upregulated compared to the other tissues (Figure 1b).

### 2.2. IL1B and IL1R1 Male Tissue Protein Localization

The immunohistochemical investigations highlighted the presence of IL1B and IL1R1 in all the male reproductive organs of the rabbits investigated. The identifiable proteins such as the molecule and its receptor were mainly observed in the cytoplasmic compartment of the lining and glandular cells with some small differences referred to the organ examined.

**Figure 2:**
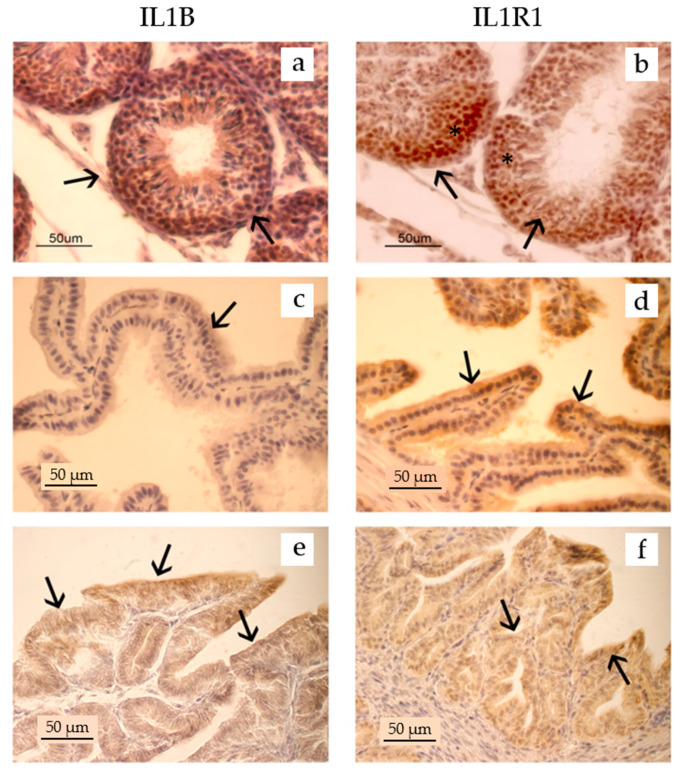
Immunopositivity for IL1B and IL1R1 in some organs of the reproductive tract of the male rabbits. Both IL1B and its receptor were observed in the cytoplasm of germinal cells, inside the convoluted seminiferous cells (**a**,**b**). Positivity is particularly evident in the basal portion of the epithelium where spermatogonia and first-order spermatocytes are found (*). In the P, a weak positivity for IL1B was observed in some secreting epithelial cells (**c**), while the positivity for the receptor was evident in a high number of the secreting epithelial cells (**d**). All glandular epithelial cells in the SV were immunopositive for IL1B (**e**) and IL1R1 (**f**). The immunopositivity for both IL1B and IL1R1 was diffused in the cytoplasm of all the epithelial cells. Sections were counterstained with hematoxylin.

In the T, the convoluted seminiferous tubules expressed a positive immunoreaction: in particular, the IL1B and IL1R1 immunopositivity was observed in the cytoplasm of the germinal cells, from the basal layer with the immature spermatogonia to the apical layers with cells that have completed the spermiohistogenesis (Figure 2a,b). The peritubular connective tissue with Leydig cells appeared negative to both IL1B and IL1R1.

In the portion of the P, the immunopositivity concerned the glandular epithelium with a strong reaction for IL1R1 in the cytoplasm of many lining cells (arrows; Figure 2d), while the positivity for IL1B was very weak and limited to a few glandular cellular elements (arrow; Figure 2c).

Finally, in the SV, the immunopositivity to both IL1B (Figure 2e) and IL1R1 (Figure 2f) was evidenced in all glandular epithelial cells.

**Figure 3:**
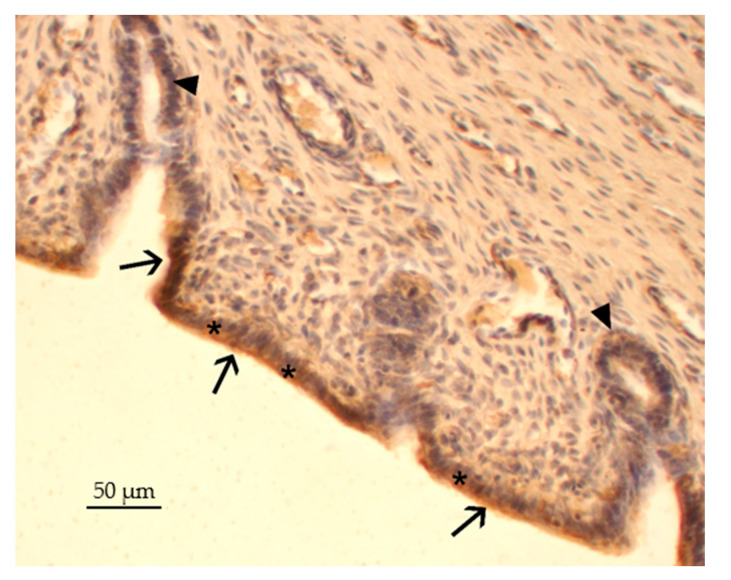
Immunopositivity for IL1R1 in the U of female rabbits. The positive immunoreaction was highlighted in the cytoplasm (*) of the lining (arrows) and glandular epithelial cells (arrows head). The section was counterstained with hematoxylin.

Regarding the immunohistochemical investigation for IL1R1 in the U, the presence of the receptor was highlighted in the cytoplasm of the lining (arrow) and glandular (arrow) epithelial cells (Figure 3).

### 2.3. IL1B Seminal Plasma Protein Expression

**Figure 4:**
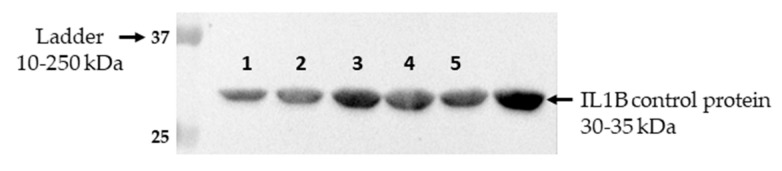
IL1B WB. The figure shows the molecular weight marker (10 to 250 kDa) on the left, only the bands corresponding to the target IL1B weights are cut. Bands 1, 2, 3, 4, and 5, positioned between 30 and 35 kDa, confirm the presence of the IL1B protein in the seminal plasma of the five rabbits involved in the experiment.

IL1B protein expression in seminal plasma was determined using WB analysis. The immunoblot showed a strong IL1B band at approximately 30–35 kDa, indicating the presence of the IL1B protein (Figure 4).

### 2.4. IL1R1 and IL1R3 Gene Expression in the Uterus

In utero, the IL1R1 and IL1R3 genes exhibited an amplification signal within the range of 25 to 30 cycles. This result is valuable as it highlights the presence of these two receptors in the uterine tissue, suggesting their functional involvement in the utero.

### 2.5. IL1B, NGF, PGF2α, and PGE2 ELISA in Uterine Cultured Model

**Figure 5:**
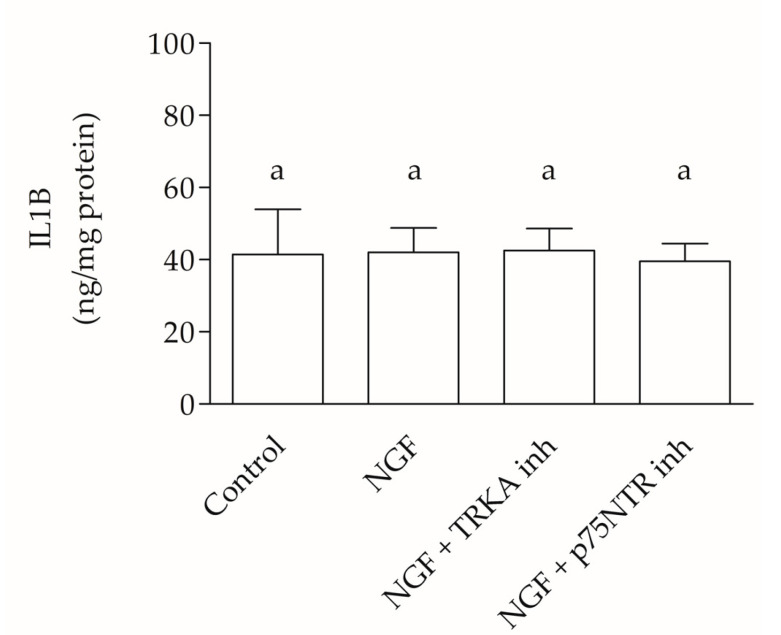
IL1B ELISA. The figure shows the IL1B levels (ng) for total protein (mg). There were no statistical differences in IL1B expression between experimental group (EG) 1 (control), EG 4 (NGF addition), EG 5 (NGF + TRKA inhibitor), and EG 6 (NGF + p75NTR inhibitor). The bar above the histograms represents the dataset standard error. Equivalent letters indicate not statistically significant differences (*p* > 0.05).

The results of the IL1B protein levels in the uterine tissue fragment (UTF) culture medium, after 2 h of incubation, obtained using the ELISA procedure, allowed for the comparison of the various experimental groups (EGs) with each other and to the control, highlighting the absence of statistically significant differences (*p* > 0.05) (Figure 5).

**Figure 6:**
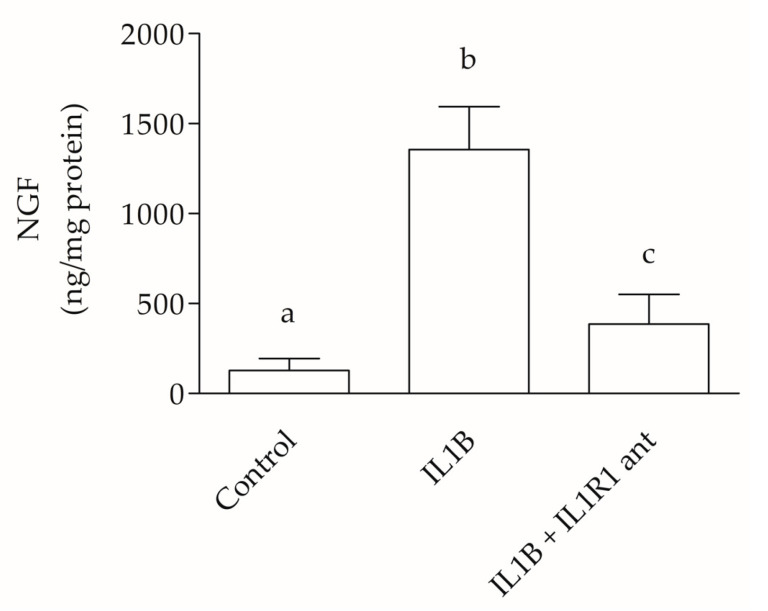
NGF ELISA. NGF protein expression levels of EG 1 (control) versus EG 2 (addition of IL1B) or versus EG 3 (addition of IL1B plus IL1R1 antagonist) show statistically significant differences. The bar above the histograms represents the dataset standard error. Different letters (a,b,c) indicate statistically significant differences (*p* < 0.05). Equivalent letters indicate not statistically significant differences (*p* > 0.05).

NGF protein levels analyzed using ELISA showed statistically significant differences between control and IL1B or control and IL1B plus IL1R1 antagonist (*p* ˂ 0.001). Similarly, protein levels indicate statistically significant differences (*p* ˂ 0.001) between IL1B and IL1B plus the IL1R1 antagonist. NGF expression in IL1B EG revealed protein overexpression (Figure 6).

**Figure 7:**
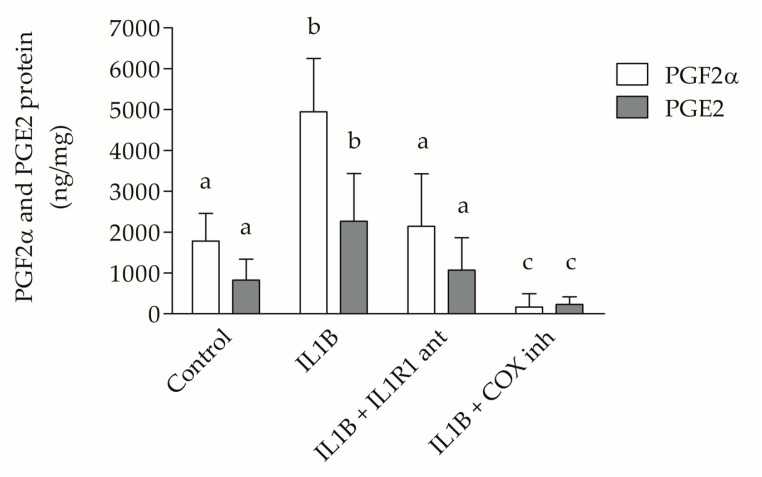
PGF2α and PGE2 ELISA. When compared with EG 2 (addition of IL1B) or with EG 7 (addition of IL1B plus COX inhibitor), the PGF2α and PGE2 expression levels in EG 1 (control) show statistically significant differences (*p* ˂ 0.001). EG 2, EG 3 (IL1B plus IL1R1 antagonist), and EG 7 when confronted with each other show statistically significant differences (*p* ˂ 0.001). Different letters (a,b,c) indicate statistically significant differences (*p* < 0.05). Equivalent letters indicate not statistically significant differences (*p* > 0.05).

PGF2α and PGE2 levels showed no significant differences when compared within the same EGs (control, IL1B, IL1B plus IL1R1 antagonist, and IL1B plus COX inhibitor). Statistical significance (*p* ˂ 0.001) was observed when PGF2α and PGE2 of the control EG were compared with PGF2α and PGE2 of the IL1B EG. A significant difference (*p* ˂ 0.001), also exists, when PGF2α and PGE2 of the control EG were compared with PGF2α and PGE2 of the IL1B plus COX inhibitor EG. PGF2α and PGE expression in IL1B EG reveals protein overexpression compared to the other EGs (Figure 7).

## 3. Discussion

This study presents the first detailed investigation of the variations in protein and mRNA expression of the IL1B/IL1R1 system in the male sex organs and U of rabbits. Our RT-qPCR analysis revealed the expression of IL1B and IL1R1 across all examined sex organs, with IHC confirming their presence in distinct patterns within the germ and somatic cells of the gonads, as well as in the epithelial and glandular cells of the P, SV, and U.

Notably, IL1B and IL1R1 showed a strong immunoreactivity in the P, particularly within luminal and glandular cells. Similarly to findings in other species, such as humans [15], the rabbit P appears to be a primary source of this cytokine for seminal plasma. It aligns with previous studies suggesting that the P gland produces large quantities of seminal plasma and prostasomes, which are crucial for enhancing sperm motility and modulating capacitation and the acrosome reaction [16]. Our data indicate a significant role for IL1B in regulating sperm behavior post-ejaculation, even though its exact impact requires further study, particularly in light of contrasting reports that associate high IL1B levels with reduced sperm motility [17,18].

Additionally, co-expression of IL1B and IL1R1 in the SV implies a potential autocrine or paracrine regulatory role in glandular function. The significant contribution of SV to seminal plasma suggests that IL1B concentration in these glands may be key for its accumulation in the reproductive fluid. Similarly, IL1B and IL1R1 were detected in the T, predominantly within the seminiferous tubules. The role of IL1B in modulating steroidogenesis in the T, as demonstrated in other species like rats and cows [19,20], is further supported by our findings of a robust IL1B expression across various germ cell stages.

Interestingly, our WB analysis revealed a prominent IL1B band in rabbit seminal plasma, highlighting potential species-specific roles for IL1B. While commonly associated with inflammation in other species [21,22], IL1B may have a unique function in rabbits, possibly related to their induced ovulation mechanism.

In female rabbits, IL1B appears to play a dual role, functioning as an inflammatory mediator as well as a modulator of reproductive processes. Previous studies suggest that IL1B is involved in luteal regression and contributes to uterine inflammation following lipopolysaccharide (LPS) treatment. Our data support these findings, as we observed that IL1B stimulated a significant increase in PGF2α and PGE2 release in uterine tissues, with COX inhibitors blocking this effect. It suggests that IL1B enhances PG production via the COX pathway, potentially influencing uterine function during the reproductive cycle.

Moreover, we found that IL1B stimulates NGF production in the U, with NGF levels rising in response to IL1B and decreasing when IL1R1 was blocked. This interaction hints at a regulatory interplay between IL1B and NGF, possibly mediated by cyclic AMP response element-binding protein (CREB) transcription factor through MAPK signaling [23,24]. These findings suggest that IL1B and NGF may synergistically regulate uterine PG production, contributing to ovarian activity modulation.

Recognizing that all research is subject to limitations in both internal and external validity, particularly when the sample size is small, we wish to emphasize that this study is intended as a preliminary investigation. To address the potential impact of a limited sample size, we plan to conduct further investigations involving a larger and different cohort. This approach will help mitigate the effects of small sample size and enhance the robustness of our findings. This research offers valuable new insights into the cytokine-mediated mechanisms underlying male–female reproductive interactions, with potential implications for advancing our understanding of reproductive regulation and improving reproductive success.

Finally, while this study stresses IL1B’s role in male–female reproductive interactions, the involvement of other cytokines in seminal plasma in stimulating the female reproductive tract remains an open area for exploration.

**Figure 8:**
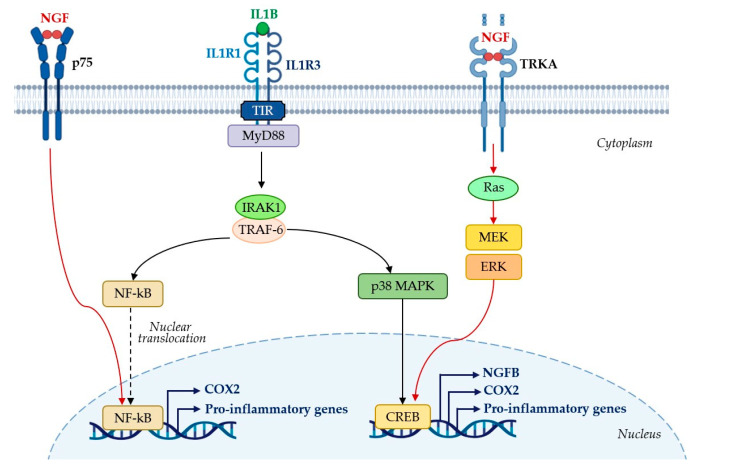
IL1B is associated with the inflammatory response and NGF production in the rabbit uterus (U). The binding of IL1B to the extracellular domain of the IL1R1 membrane receptor allows the recruitment of the IL1R3 co-receptor. This interaction forms the IL1R1/IL1R3 heterodimer via the intracellular toll–interleukin-1 receptor (TIR) domains of the two receptor polypeptide chains, thereby initiating signal transduction. The IL1R1/IL1R3 receptor complex then recruits the adaptor protein myeloid differentiation primary response gene 88 (MyD88), which activates interleukin-1 receptor-associated kinase 1 (IRAK1) and tumor necrosis factor receptor-associated factor 6 (TRAF-6). This activation cascade stimulates the nuclear factor-kappa B (NF-κB) and p38 mitogen-activated protein kinase (MAPK) signaling pathways. Upon activation, NF-κB translocates to the nucleus where it acts as a transcription factor, upregulating the gene expression of pro-inflammatory cytokines (e.g., IL-1, IL-6, and TNFα) and COX2 which is essential for the synthesis of PGE2 and PGF2α starting from the arachidonic acid. Similarly, IL1B-mediated activation of p38 MAPK induces the transcription factor cyclic AMP response element-binding protein (CREB), which promotes the transcription of COX2, pro-inflammatory cytokine, and NGF gene, explaining how the inflammatory cytokine IL1B enhances NGF production in the rabbit U. In addition to IL1B, NGF, through its receptors, p75NTR and TRKA, activate NF-κB and Ras-Raf-MEK-ERK-CREB signaling (red arrows), contributing to COX2 transcription and as a consequence prostaglandins production in the U.

Figure 8 shows a proposed interaction between IL1B, the inflammatory response, and NGF production in the rabbit U.

## 4. Materials and Methods

### 4.1. Animal Enrollment and Experimental Design

This research was carried out following a revised protocol approved by the Bioethics Committee of the University of Perugia (prot. no. 365301 dated 12 October 2023), according to welfare guidelines and principles for animal protection. Adult New Zealand white rabbits, aged 5–8 months and weighing 4.5–5 kg, were used in all experimental steps. The environmental housing conditions were monitored because rabbits are highly susceptible to environmental stress. Rabbits were maintained in a controlled condition: temperature ranged from 17 to 22 °C, relative humidity was 60% and a continuous photoperiod of 16 h light per day was maintained. Fresh water was always available [25]. The rabbits were fed with commercial pelleted food.

The experimental procedure was divided into two steps.

Step 1: Male

- IL1B and IL1R1 gene expression and tissue protein localization

In this step, the tissues of the male reproductive system, in particular the T, P, and SV of rabbits, were studied. In T, P, and SV, the expression of ILB1 and IL1R1 genes was investigated using RT-PCR, while the ILB1 and IL1R1 protein expression was observed through IHC. In seminal plasma, the presence of IL1B protein was evaluated in WB.

Step 2: Female

- IL1R1 and IL1R3 gene expression and protein tissue localization
- Functional model of molecular reproduction crosstalk

**Figure 9:**
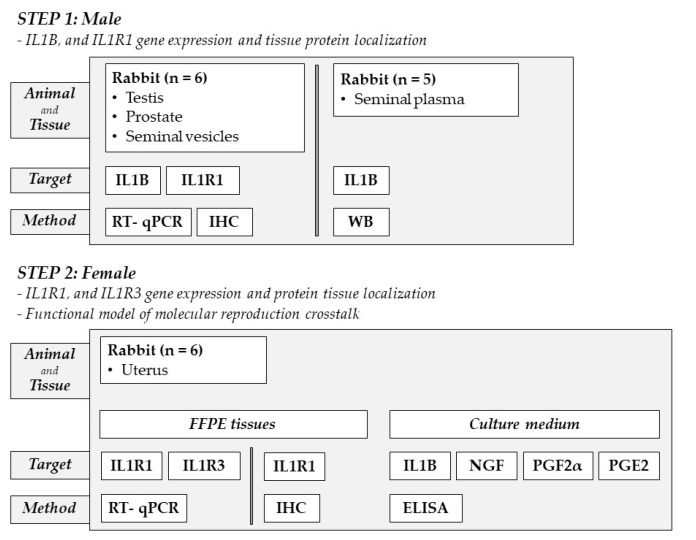
The experimental procedure consists of two steps. Step 1 involved the investigation of IL1B and IL1R1 gene (RT-PCR) and protein (IHC) expression in reproductive tissues (T, P, and SV) in six male rabbits. Protein IL1B evaluation (WB) in the seminal plasma of 5 rabbits was included in this step. In Step 2, the U of six rabbits sliced into small pieces was incubated in vitro for two hours, and then the concentration of IL1B, NGF, PGE2, and PGF2α was assessed in the culture medium using ELISA method. The U (n = 6) evaluation of IL1R1 and IL1R3 gene (qPCR) and IL1R1 protein (IHC) were included in this step.

In this step, we investigated IL1R1 and co-receptor IL1R3 gene expression using RT-qPCR in Formalin-fixed paraffin-embedded (FFPE) U. The IL1R1 protein location was also investigated using IHC. In addition, the female molecular crosstalk was observed in vitro by incubating the UTF with target ligands sole or with their cognate receptor antagonist inhibitors. After incubation, the release of NGF, IL1B, PGF2α, and PGE2 in the medium was assessed using ELISA procedure to study the changes in the microenvironment (Figure 9).

In Step 1, six male rabbits were sacrificed for T, P, and SV tissue sampling, while seminal plasma was collected from five rabbits. In Step 2, six female rabbits were sacrificed for uterine sampling.

### 4.2. Collection and Processing of Tissue and Seminal Plasma

The rabbits were sacrificed using cervical dislocation following the guidelines and principles for the care and use of research animals. Upon sacrifice, sex organs (male: T, P, and SV; female U) were promptly removed and thoroughly washed with RNAse-free buffered saline solution (PBS) and then frozen at −80 °C until use. Samples for IHC procedure were quickly dipped in 10% neutral-buffered formalin solution in PBS (0.1 M, pH 7.4), left for 36 h, and then processed until the paraffin wax embedding. Seminal plasma was collected from live animals by artificial vaginal. To evaluate sperm count and sperm morphological integrity, 300 cells/animal were analyzed using light microscopy. The sperm was frozen at −80 °C up to the time of use.

### 4.3. IL1B and IL1R1 Male Tissue RT-PCR

Total RNA from freshly collected T, P, and SV reproductive tissues of rabbits (n = 6) was purified using a NucleoSpin RNA Extraction kit (MN-740955; D-Mark Biosciences, Macherey-Nagel, Bethlehem, PA, USA). After Deoxyribonuclease I (DNAase I Amp. Grade) treatment, the purified total RNA was retrotranscripted with Superscript III reverse transcriptase (Superscript III First-Strand Synthesis System). RNA quality and quantity were assessed by spectrophotometry (NanoDrop™ 2000/2000c, Thermo Fisher Scientific, Waltham, MA, USA) and fluorometry (Qubit RNA As-say, Life Technologies, Waltham, MA, USA). Total RNA was reverse transcribed in 20 μL iSCRIPT cDNA (Bio-Rad, Hercules, CA, USA) according to the manufacturer’s recommendations. Controls without reverse transcriptase (RT-) were included to check for genomic DNA contamination.

QPCR amplification was executed in a final volume of 20 μL using 10 μL of SsoAdvanced Universal SyBrgreen Supermix (Bio-Rad, Hercules, CA, USA), 1 μL of cDNA (diluted 1:10), 1 μL of primer. Primers were designed using Primer-BLAST (NCBI) and were synthesized using Life Technologies (Monza, taly). Primer characteristics are listed in Table 1.

The primer dissociation peak was estimated with the melting curve: 95 °C for the 15th, 60 °C for the 20th followed by ramping to 95 °C with fluorescence measurement every 2.5 °C. QPCR amplification reactions were run in a 96-well optical plate on StepOne Plus Real-time PCR instrument (Applied Biosystems). Three technical replicates were performed for each biological sample, and the average Cq value (quantification cycle according to MIQE guidelines [26]) was calculated. Amplification signals and Cq values were determined using StepOne Software v2.3 (Applied Biosystems, Foster, CA, USA). No template controls were enrolled in the qPCR to control possible genomic DNA contamination. The mRNA target expression levels were estimated using the Livak 2^−ΔCq method [27], using 18S gene to normalize target gene expression.

### 4.4. IL1B and IL1R1 Male Tissue IHC

Tissue samples (T, P, and SV) of male rabbits (n = 6) were fixed, dehydrated, cleared, embedded in paraffin wax, and then cut in 5 μm-thick serial sections. After morphological evaluation in hematoxylin–eosin solution to exclude pathologies, IHC was performed in all tissues. FFPE sections, after being dewaxed and hydrated, were heated to reveal the antibody epitopes. The endogenous peroxidase was blocked with a 3% hydrogen peroxide solution (10 min), and thus, to exclude non-specific bindings, the sections were incubated (30 min) with normal goat serum. Subsequently, to perform the IHC, the sections were incubated overnight (ON) at room temperature with IL1B and IL1R1 antibodies. The best dilution to obtain a signal intensity without background was used for each primary antibody. The next day, sections were incubated with a biotin-conjugated secondary antibody for 30 min. The binding sites were detected using the DAB chromogen (DAB substrate kit, Vector Laboratories, Burlingame, CA, USA). Antibody specifications and dilution are provided in Table 2.

### 4.5. IL1B WB

WB was performed in rabbit male (n = 5) seminal plasma. The protein amount in the seminal plasma was quantified spectrophotometrically using the dye-binding method based on the Bradford assay (Bio-Rad Protein Assay Dye Reagent Concentrate, 5000006, Bio-Rad, Hercules, CA, USA). A 40 µg of proteins, diluted in sample buffer, was loaded in a 10% sodium dodecyl sulfate polyacrylamide gel (SDS-PAGE) and separated based on their size. The proteins were then transferred onto nitrocellulose membranes using Trans-Blot Turbo Transfer System (Bio-Rad, Hercules, CA, USA) before blocking and incubation with primary antibodies. The primary antibody against IL1B (Mouse IL-1 beta/IL-1F2, AF-401-NA Biotechne, R&DS System, Minneapolis, USA) was incubated ON at 4 °C. The membranes were rinsed in tris-buffered saline (TBS) with tween, and incubated with the secondary horseradish peroxidase (HRP)-labeled antibody for 1 h at room temperature under gentle agitation. The membranes were washed 4 times (10 min) before detecting the immune complexes using a chemoluminescent substrate (Euroclone, Life Science Division, Siziano, Italy). Densitometric analysis was performed with ImageLab software 3.0 (Bio-Rad, Hercules, CA, USA).

### 4.6. IL1R1 and IL1R3 Uterine RT-qPCR

Total RNA was extracted from three 5 µm-thick FFPE sections of rabbit U using the FFPE RNA Purification Kit (Norgen Biotek Corp., Thorold, ON, Canada). During RNA isolation, samples were treated with DNase I provided in the Extraction Kit to remove on-column DNA. RNA quantification and cDNA synthesis were performed as described in Step 1.

To increase the sensitivity of qPCR analysis, a pre-amplification reaction was carried out using 3 μL of cDNA, 1 μL of TaqMan Gene Expression Assays (Table 3), 10 μL of SsoAdvanced™ Universal Probes Supermix (Bio-Rad Laboratories, Hercules, CA, USA), and water up to 20 μL. Pre-amplification reactions were run for 3 min at 95 °C, 13 cycles of 15 s at 95 °C, and 4 min at 58 °C.

For Real-time qPCR, TaqMan probes were used in place of primers to improve the specificity of the amplification signal. The reaction was performed using 1.5 μL of pre-amplification reaction, 1 μL of TaqMan probes (Table 3), and 10 μL of SsoAdvanced™ Universal Probes Supermix (Bio-Rad Laboratories, Hercules, CA, USA), in a final volume of 20 μL. The qPCR conditions were initial activation 30 s at 95 °C and 2-step cycling (denaturation 5 s at 95 °C, annealing/extension 5 s at 60 °C) for a total of 40 cycles.

Both pre-amplification and qPCR amplification reactions were run in a 96-well optical plate on StepOne Plus Real-time qPCR instrument (Applied Biosystems). RT-controls were enrolled in the qPCR to check for potential genomic DNA contamination. Amplification signals were computed and Cq values were determined using StepOne Software v2.3 (Applied Biosystems).

### 4.7. IL1R1 Female Tissue IHC

Tissue samples (U) of female rabbits (n = 6) were fixed, dehydrated, cleared, embedded in paraffin wax, and then cut into 5 μm-thick serial sections. The entire subsequent procedure follows what has already been indicated in Section 4.4.

### 4.8. Ex Vivo Uterine Tissue Cultured Model

Postmortem female rabbit U (n = 6) was extracted, rinsed with PBS, and then cut with fine surgical scissors (21 pieces per rabbit, each weighing approximately 30 mg). UTF were randomly distributed (1 UTF per well) with 1 mL of culture medium 199 plus Earles Balanced Salt Solution, 2.2 mg/mL sodium bicarbonate, 2.3 mg/mL HEPES, and 3% BSA (*w*/*v*). All reagents were obtained from GIBCO (Grand Island, NY, USA).

The 24-well culture plates purchased from Becton Dickinson Co. (Clifton, NJ, USA) were incubated for 4 h (37 °C in 5% CO_2_). After the end of the incubation time, the medium was collected separately to be stored at −20 °C for further ELISA determination.

The experimental in vitro tissue slice culture model consists of seven EGs. The EGs were tested in triplicate and each EG includes culture medium and UTF. The experimental design of the 7 EGs was described below: (1) Control (medium and UTF alone); (2) Recombinant Human IL1B, R&D Systems (Minneapolis, MN, USA); (3) IL1B + IL1B receptor antagonist, Sobi Kineret^®^ (Swedish Orphan, Stockholm, Sweden); (4) Human NGF, Sigma-Aldrich (St. Louis, MO, USA); (5) NGF + TRKA Inhibitor, catalogue reference GW 441756, Tocris (Milano, Italy); (6) NGF + p75NTR Inhibitor, catalogue reference PD 90780, Tocris (Milano, Italy); (7) IL1B + COX inhibitor, Sigma-Aldrich (St. Louis, MO, USA).

Based on a pilot study, we selected the specific design and the minimum efficacy dose for the NGF, IL1B, IL1R1 antagonist, TRKA Inhibitor, and p75NTR Inhibitor (Table 4).

### 4.9. IL1B, NGF, PGF2α, and PGE2 Evaluation Using ELISA

The analytical procedure used to assess the expression of IL1B, NGF, PGF2α, and PGE2 in the culture medium was the ELISA approach. The ELISA kits used for PGF2α and PGE2 (catalog reference ADI-901-069 and ADI-901-001, respectively) were supplied by Enzo Life Science Inc. (Farmingdale, NY, USA). Human NGF and IL1B proteins (catalog reference DY256 and E-EL-RB0013, respectively) were analyzed using ELISA kits supplied by R&D Systems (Minneapolis, MN, USA). All the recommended procedures provided by the manufacturer were followed in performing the ELISA procedures. Pilot linearity experiments were performed before setting up the ELISA to determine the optimal culture medium concentration. In all plates, in addition to the standard curve, an aliquot serum sample was run for result normalization and comparison between plates. All samples were run in triplicate and the mean values were used for data analysis. These kits contained a pre-coated ELISA plate with the specific capture antibody for the rabbit analyte (PGF2α, PGE2, IL1B, and Human NGF). The culture medium (100 μL) was added to ELISA plate wells to react with the specific antibody. The plate was incubated for 120 min at 37° C. After three washes, the specific detection antibody Avidin–HRP conjugate (100 μL) was added and incubated (20 min). After three washes, the substrate solution (90 μL) was added to each well, and the plate was incubated 20 min at room temperature. Only those wells that contain Rabbit analyte, biotinylated detection antibody, and Avidin–HRP conjugate will appear blue. The enzyme–substrate reaction was terminated by adding a stop solution (50 μL). All incubations were performed at room temperature. The optical density (OD) was measured spectrophotometrically at a wavelength of 450 and 570 nm (Tecan Spark, Tecan Group Ltd., Männedorf, Switzerland). The OD value were proportional to the concentration of the analyte. The concentrations of all analytes found in the wells were calculated by comparing the OD of the samples with the standard curve (Figure 2).

### 4.10. Statistical Analysis

One-way analysis of variance (ANOVA) and Kruskal–Wallis test were used to test differences in IL1B and IL1R1 RT-PCR gene expression and IL1B and NGF ELISA protein concentration. Two-way ANOVA and Bonferroni multiple comparisons were used to evaluate differences in PGF2α and PGE2 ELISA concentrations in different tissue culture models. ANOVA analysis was performed using GraphPad Prism 9 software.

## 5. Conclusions

This study provides preliminary evidence that IL1B and its receptor IL1R1 are present in rabbit male reproductive tissues (T, P and SV) and seminal plasma at both the gene and the protein levels, supporting IL1B’s involvement in reproductive regulation. Notably, IL1B and its receptors, IL1R1 and co-receptor IL1R3, are also expressed in rabbit U, where IL1B enhances the inflammatory response and promotes PG synthesis through COX activity. Our findings suggest that IL1B directly influences uterine NGF secretion, while NGF does not affect IL1B production, indicating that NGF may be produced in response to seminal plasma mediators like IL1B. Future research should explore the roles of PGF2α in mediating IL1B’s effects on uterine NGF production and investigate other ovulation-inducing factors in seminal plasma. Further understanding of these interactions could improve fertility management strategies in rabbits and potentially in other mammals with both induced and spontaneous ovulation.

## Figures and Tables

**Table 1:** PCR primer. The table lists the gene name (acronym), the sequence identification number, the forward (F) and reverse (R) primers, and the amplicon length in the base pair (bp).

Gene	Sequence Number	Primers	bp
IL1B	NM_001082201.1	F—TGAGGCCGATGGTCCCAATTA	183
		R—AAGGCCTGTGGGCAGGGAAC	
IL1R1	XM_008253215.2	F—CTGCTGTCTTGGCCCTGTTA	137
		R—GCATCCTCTTGAAAGGCCCT	
18S		QuantumRNA™ 18S endogenous reference gene	489

**Table 2:** Antisera characteristics and dilutions. The table indicates the antibody name, the species in which the antibody was raised, and the working solution.

Antisera	Host	Dilution
^1^ Polyclonal anti-IL1B B	Rabbit	1:500
^2^ Polyclonal anti IL1R1	Rabbit	1:500
^3^ Anti-rabbit IgG Biotin conjugated	Goat	1:200

^1^ Anti—IL1B, catalog reference GTX74034 (GeneTex, Irvine, CA, USA). ^2^ Polyclonal anti-IL1R1, catalog reference 106278 (Abcam Cambridge, UK). ^3^ Anti-rabbit Biotin conjugated catalog reference BA-1000–1.5 (Vector Laboratories, Newark, CA, USA).

**Table 3:** TaqMan Gene Expression probes. The table shows the gene acronym, the TaqMan probe ID, the reference sequence, the exon boundary, and the amplicon length (bp). The selected TaqMan probes were designed for *Oryctolagus cuniculus* sequences.

Gene Symbol	TaqMan ID	Reference Sequence	Exon Boundary	Amplicon bp
IL1R1	Oc06785929_m1	XM_002709884.3	8–9	76
IL1R3	Oc06776406_m1	XM_008266655.2	2–3	65

**Table 4:** Plate well design. The table shows the EGs from 1 to 7 (first column on the left) and the corresponding ligands (IL1B and NGF), or receptor antagonists (IL1R1) or inhibitors (TRKA, p75NTR, and COX inhibitors) used in the respective EG (when in wells added as +). Each column indicates the working solution and the reagent provider (as a note).

EGs	IL1B ^1^ 100 mg/Well	IL1R1 Ant ^2^ 7 mg/Well	NGF ^3^ 8.1 ng/Well	TRKA Inh ^4^ 10 pg/Well	p75NTR Inh ^5^ 10 pg/Well	COX Inh ^6^ 85 pg/Well
1	-	-	-	-	-	-
2	+	-	-	-	-	-
3	+	+	-	-	-	-
4	-	-	+	-	-	-
5	-	-	+	+	-	-
6	-	-	+	-	+	-
7	+	-	-	-	-	+

^1^ Recombinant Human IL1B, R&D Systems (Minneapolis, MN, USA). ^2^ IL1B receptor antagonist, Sobi Kineret^®^ anakinra (Swedish Orphan, Stockholm, Sweden). ^3^ Human NGF, Sigma-Aldrich (St. Louis, MO, USA). ^4^ TRKA Inhibitor, Tocris catalog reference GW 441756 (Milano, Italy). ^5^ p75NTR Inhibitor, Tocris catalog reference PD 90780 (Milano, Italy). ^6^ COX nonselective cyclooxygenase inhibitor (acetylsalicylic acid) Sigma-Aldrich (St. Louis, MO, USA).

## Data Availability

All data are contained within the article.

## Abbreviations

ANOVA	Analysis of variance
COX2	Cyclooxygenase-2
CREB	cyclic AMP response element-binding protein
EG	Experimental group
ELISA	Enzyme-linked immunosorbent assay
FFPE	Formalin-fixed paraffin-embedded
HRP	Avidin–Horseradish Peroxidase
IHC	Immunohistochemistry
IL1	Interleukin-1
IL1A	Interleukin-1 A
IL1B	Interleukin-1 B
IL1R1	Interleukin-1 receptor type 1
IL1R3	Interleukin 1 receptor accessory protein
IL6	Interleukin-6
IRAK1	Interleukin-1 receptor-associated kinase 1
LPS	Lipopolysaccharide
MAPK	Mitogen-activated protein kinases
Myd88	Myeloid differentiation primary response 88
NF-kB	Nuclear factor-kappa B
NGF	Nerve growth factor
OD	Optical density
ON	Overnight
P	Prostate
p75NTR	p75 neurotrophin receptor
PBS	Buffered saline solution
PG	Prostaglandin
PGE2	Prostaglandin E2
PGF2α	Prostaglandin F2α
RT-qPCR	Quantitative reverse transcription PCR
RT	Reverse transcription
SDS-PAGE	Sodium dodecyl sulfate polyacrylamide gel
SV	Seminal vesicles
T	Testis
TBS	Tris-buffered saline
TIR	Toll-interleukin-1 receptor
TNF	Tumor necrosis factor
TRAF-6	Tumor necrosis factor receptor-associated factor 6
TRKA	Tropomyosin receptor kinase A
U	Uterus
UTF	Uterine tissue fragment
WB	Western Blotting
